# Dietary compounds in modulation of gut microbiota-derived metabolites

**DOI:** 10.3389/fnut.2022.939571

**Published:** 2022-07-19

**Authors:** Wuwen Feng, Juan Liu, Hao Cheng, Dandan Zhang, Yuzhu Tan, Cheng Peng

**Affiliations:** ^1^State Key Laboratory of Southwestern Chinese Medicine Resources, School of Pharmacy, Chengdu University of Traditional Chinese Medicine, Chengdu, China; ^2^Key Laboratory of the Ministry of Education for Standardization of Chinese Medicine, Chengdu University of Traditional Chinese Medicine, Chengdu, China

**Keywords:** dietary compounds, gut microbiota-derived metabolites, short-chain fatty acids, bile acids, trimethylamine, branched-chain amino acids, tryptophan and indole derivatives

## Abstract

Gut microbiota, a group of microorganisms that live in the gastrointestinal tract, plays important roles in health and disease. One mechanism that gut microbiota in modulation of the functions of hosts is achieved through synthesizing and releasing a series of metabolites such as short-chain fatty acids. In recent years, increasing evidence has indicated that dietary compounds can interact with gut microbiota. On one hand, dietary compounds can modulate the composition and function of gut microbiota; on the other hand, gut microbiota can metabolize the dietary compounds. Although there are several reviews on gut microbiota and diets, there is no focused review on the effects of dietary compounds on gut microbiota-derived metabolites. In this review, we first briefly discussed the types of gut microbiota metabolites, their origins, and the reasons that dietary compounds can interact with gut microbiota. Then, focusing on gut microbiota-derived compounds, we discussed the effects of dietary compounds on gut microbiota-derived compounds and the following effects on health. Furthermore, we give our perspectives on the research direction of the related research fields. Understanding the roles of dietary compounds on gut microbiota-derived metabolites will expand our knowledge of how diets affect the host health and disease, thus eventually enable the personalized diets and nutrients.

## Introduction

Gut microbiota is a group of microorganisms including bacteria, archaea, eukarya, etc., that dwell in the human gastrointestinal tract (1). The number of microorganisms in human gut microbiota is estimated to be as many as 10^14^ bacterial cells, which is 10 times larger than the number of human cells (2). In recent decades, numerous studies have demonstrated that gut microbiota plays important role in maintenance of health by acting as a barrier to defend against pathogens, maintaining the integrity of the epithelial barrier, modulating the metabolism and immune functions of hosts, communicating with central nervous system (3). On the contrary, gut microbiota dysbiosis can lead to a large number of diseases, such as non-alcoholic fatty liver disease, obesity, inflammatory bowel disease, cancer, allergy, and depression (2, 4). Because the importance of gut microbiota in health and disease, gut microbiota has become an important research frontier in health-related research fields.

Normally, the gut microbiota is confined within the gastrointestinal lumen by gut barrier and translocation of gut bacteria can lead to local and systematic inflammation (5). To overcome the spatial limitation, gut microbiota develops a tactic of releasing a large number of gut microbiota metabolites from different chemical classes to exert extensive effects on host organs that are near or far away from gastrointestinal lumen (6). Gut microbiota contains 5 × 10^6^ unique microbial genes collectively, which outnumber the genes of humans (7). With these genes, gut microbiota is capable of synthesizing a large group of metabolites. These gut microbiota-derived metabolites include, but not limited to, short chain fatty acids (SCFAs), bile acids, vitamins, tryptophan and indole derivatives (8). These metabolites can exert the functions of regulating of gut microbiota composition, host metabolism, nutrition absorption, gut motility, local and systemic immune response, circadian rhythm, etc., to maintain the health or promote the development of diseases (6).

Diet is one of the most important regulators of gut microbiota. Many dietary compounds exhibit low bioavailability or cannot not be absorbed directly, such as polysaccharides and polyphenol (9). When orally taken, these compounds can pass the small intestine and enter the colon, a comfortable place that most gut bacteria live. In the colon, dietary compounds can interact with gut microbiota. On one side, gut microbiota can transform the food compounds and produce new compounds derived from dietary compounds. For example, rutin can be transformed to quercetin and further into protocatechuic acid, 3,4-dihydroxyphenyl-acetic acid, etc., by gut microbiota (10). On the other side, food compounds can induce the functional and compositional change of gut microbiota. For example, coffee and its major components caffeine and chlorogenic acid can alter gut microbial community and SCFAs levels (11). The gut microbiota transformation of dietary compounds and dietary compounds modulation of gut microbiota composition have been extensively reviewed (12–14), however, there is no focused review on the effects of dietary compounds on gut microbiota metabolites. In this review, we focus on the effects of dietary compounds on gut microbiota-derived compounds and the following effects on health.

## Gut microbiota metabolites in a nutshell

Gut microbiota not only contain a large number of bacteria that belong to more than 1,000 species, it also contain millions of microbial genes that is 150-fold larger than the number of human gene complements (7). With the aid of these genes, gut microbiota is capable of producing a large number of enzymes. These enzymes can ferment diverse food compounds that are not digested by human enzymes such as fiber or compounds that are released by human body such as primary bile acids. Consequently, gut microbiota can synthesis and release a large number of metabolites with great diverse of chemical structures. These gut microbiota-derived metabolites can be broadly grouped into 3 categories: (I) metabolites that are produced directly from dietary compounds by gut microbiota, such as SCFAs, tryptophan, and indole derivatives of tryptophan; (II) metabolites that are originally synthesized by hosts and chemically transformed by gut microbiota, such as secondary bile acids; (III) metabolites that are produced by gut microbiota *de novo* (15). A lot of metabolites show similar structure and bioactivities, and they are therefore grouped together. Currently, the extensively studied gut microbiota-derived metabolites include SCFAs, bile acids, branched chain amino acids (BCAAs), tryptophan and indole derivatives, etc. In Table 1, we have listed the typical groups of gut microbiota metabolites and their functions.

**Table 1:** Typical gut microbiota metabolites and their functions in health and disease.

**Typical metabolites**	**Typical targets**	**Specific functions**	**Typical diseases associated**	**References**
Short-chain fatty acids (SCFAs) (acetate, propionate, butyrate, isovalerate, isobutyrate, valerate, etc.)	G-protein-coupled receptors (GPR41, GPR43, GPR109A, GPR81, GPR91) and nuclear class I histone deacetylases (HDAC1 and HDAC3)	Regulate gut barrier, appetite, energy metabolism, gut hormones; reduce proinflammatory cytokines; modulate systemic immune response	Diabetes, obesity, non-alcoholic fatty liver disease, hypertension, atherosclerosis, ulcerative colitis, Crohn's disease, colorectal cancer, autism spectrum disorder, Parkinson's disease, asthma	(6, 16–18)
Bile acids (BAs) (ω-muricholic acid, murideoxycholic acid, deoxycholic acid, lithocholic acid, hyodeoxycholic acid, etc.)	Farnesoid X receptor (FXR), Takeda G-protein receptor 5 (TGR5), vitamin D3 receptor (VDR), pregnane X receptor/steroid and xenobiotic-sensing receptor (PXR/SXR), constitutive androstane receptor (CAR), etc.	Facilitate lipids absorption; regulate gut microbiota composition, intestinal immunity, gut motility, lipid and glucose homeostasis, amino acid metabolism	Primary biliary cholangitis, obesity, non-alcoholic fatty liver disease, atherosclerosis, ulcerative colitis, cancer, Alzheimer's disease, Parkinson's disease	(19–21)
Trimethylamine (TMA), and indirect product trimethylamine-N-oxide (TMAO)	Nuclear factor-κB (NF-κB), protein kinase C (PKC), and nucleotide-binding oligomerization domain–like receptor family pyrin domain–containing 3 (NLRP3) inflammasome	Promote inflammation, thrombosis; influences myocardial hypertrophy and fibrosis; promotes mitochondrial dysfunction	Non-alcoholic fatty liver disease, diabetes, heart failure, obesity, atherosclerosis, hypertension	(22–24)
Branched-chain amino acids (BCAAs) (leucine, isoleucine, and valine)	Mammalian target of rapamycin complex 1 (mTORC1), glutamate dehydrogenase	Acting as building blocks for all life, especially involved in protein synthesis and insulin secretion	Insulin resistance, type 2 diabetes, cardiovascular diseases, cancers	(25, 26)
Tryptophan and indole derivatives (indole-3-lactic acid, indole acetic acid, indole-3-acetamide, etc.)	Aryl hydrocarbon receptor (AhR) and PXR	Regulate gut barrier, gut motility, gut hormone secretion, and systemic immune response	Ulcerative colitis, Crohn's disease, irritable bowel syndrome, obesity, Alzheimer's disease, Parkinson's disease, schizophrenia,	(27–29)
Gases (H_2_S, H_2_, CO_2_, CH_4_, NO)	NO acts on soluble guanylate cyclase	CH_4_ modulates gut motility; H_2_S regulates epithelial secretion, gut inflammation, and susceptibility to infections; NO regulates blood flow	Parkinson's disease, colitis, ulcer	(30–33)
Others (lipopolysaccharides; vitamins such as vitamin B2, organic acids such as benzoate; polyamines such as cadaverine; neurotransmitters such as dopamine)	Lipopolysaccharides acts on CD14/Toll-like receptor 4, vitamins act on vitamin receptors, neurotransmitters act on adrenergic receptors	Influence gut barrier; regulate intestinal or systemic immune reaction; act as the nutrients; be toxic to host cells	Insulin resistance, obesity, type 2 diabetes mellitus, non-alcoholic fatty liver disease, chronic hepatitis C irritable bowel syndrome, ulcerative colitis	(4, 34–36)

After the production and releasing, gut microbiota metabolites can be absorbed and transferred into circulating system. It is estimated that gut microbiota metabolites can account for 10% of the total metabolites in blood of mammalian (37). When absorbed, gut microbiota metabolites can be transported to target organs and tissues that are remote from gastrointestinal tract, and therefore exert a wide range of activities on hosts. These activities include, but not limited to, regulating the composition and function of gut microbiota, acting as nutrition, influencing nutrition absorption, modulating host metabolism, influencing intestinal barrier and gut motility, impacting the local and systemic immune response, modulating circadian rhythm and nervous system (6). With these bioactivities, gut microbiota metabolites play important roles in the development and progress of a variety of diseases such as cancer, non-alcoholic fatty liver disease, hypertension, Parkinson's disease, and ulcerative diseases (30, 38–40).

## Dietary compounds can interact with gut microbiota and modulate gut microbiota metabolites

In gastrointestinal tract, most of the dietary compounds can be digested and absorbed by human body. Even so, significant amounts of digestible dietary compounds can reach the colon. For instance, the majority of proteins can be digested and absorbed in the small intestine, a relatively large amounts of proteins and amino acids (about 6–18 g per day) can reach the colon (41). In addition, the bioavailability of some types of dietary compounds is very low and thus these compounds can reach the colon as well. For example, the bioavailability of polyphenols is very poor and is often <10% (42). Thus, the unabsorbed dietary compounds such as polyphenols and amino acids can enter the colon. The colon is an ideal place for the interactions between gut microbiota and dietary compounds because colon alone contains over 70% of all the microbes in the human body including the surface of body (2). Other reasons that render colon an ideal place also include the suitable pH and the long time that enable the direct contact between gut microbiota and dietary compounds. The direct interactions between dietary compounds and gut microbiota include (I) gut microbiota can transform the dietary compounds directly; (II) dietary compounds can modulate the composition of gut microbiota; (III) dietary compounds can modulate the metabolites of gut microbiota. In addition to direct reactions, dietary compounds can also interact indirectly with gut microbiota *via* modulation of gastrointestinal pH, gastrointestinal transit time, the synthesis and release of immune materials such as antimicrobial peptides and secretory immunoglobulin A (43). For example, dietary compounds such as polyphenols and peptides can modulate the gastrointestinal pH, which can further modulate the composition of gut microbiota and the catalytic activity of enzymes (42, 44, 45). Whatever the indirection, the final reactions of indirect reactions can be only attributed to the types of direct reactions we discussed.

The bioavailability of many dietary compounds and herbal compounds is very low, yet they possess strong bioactivities. This discrepancy has perplexed researchers for a long time (46). Studies in recent decade have revealed that the interaction between gut microbiota and these compounds is one of the keys to decipher this conundrum. Gut microbiota can transform the chemical structure of dietary compounds and thus improve the bioavailability or potency. For example, curcumin, a polyphenolic compound with high hydrophobicity and poor solubility, exhibits wide spectrum of pharmacologic effects on diseases such as inflammatory diseases, cancers, cardiovascular diseases, and metabolic diseases (47). Animal studies and clinical trials indicated that the bioavailability of curcumin is very poor (about 1%) (48). Recent studies showed that gut microbiota can transform the curcumin into ferulic acid, dihydrocurcumin, tetrahydrocurcumin, curcumin-L-cysteine, bisdemethylcurcumin, etc., *via* gut bacteria such as *Blautia* sp. MRG-PMF1, *Bacillus megaterium* DCMB002 (49, 50). Some of these products, such as dimethoxycurcumin, have superior bioactivities compared with their parent compound curcumin (51). Thus, the biotransformation of curcumin by gut microbiota can explain the contradiction between low bioavailability and strong bioactivities of curcumin. In addition to biotransformation, the gut microbiota metabolites may also explain this contradiction as well. Hydroxysafflor yellow A (HSYA) is a water-soluble compound isolated from *Carthamus tinctorius* L. with only 1.2% oral bioavailability (52). Yet it shows a group of bioactivities such as anti-tumor, neuroprotective, hepatoprotective, and pulmonary protective effects (53). In a high-fat diet-induced obese mice, oral administration of HSYA increased the abundances of *Akkermansia* and *Romboutsia*, as well as SCFAs-producing bacteria *Butyricimonas* and *Alloprevotella* (54). In addition, HSYA significantly increased acetic acid, propionic acid, and butyric acid, a group of SCFAs that is directly associated with obesity and intestinal integrity when their levels are decreased (54). The study suggested that increase of SCFAs may contribute to the pharmacological effects of HSYA. Taken together, transformation of dietary compounds by gut microbiota and modulation of gut microbiota metabolites by dietary compounds can explain the contradiction between low bioavailability and strong bioactivities of some dietary compounds.

## Gut microbiota metabolites modulated by dietary compounds

**Figure 1:**
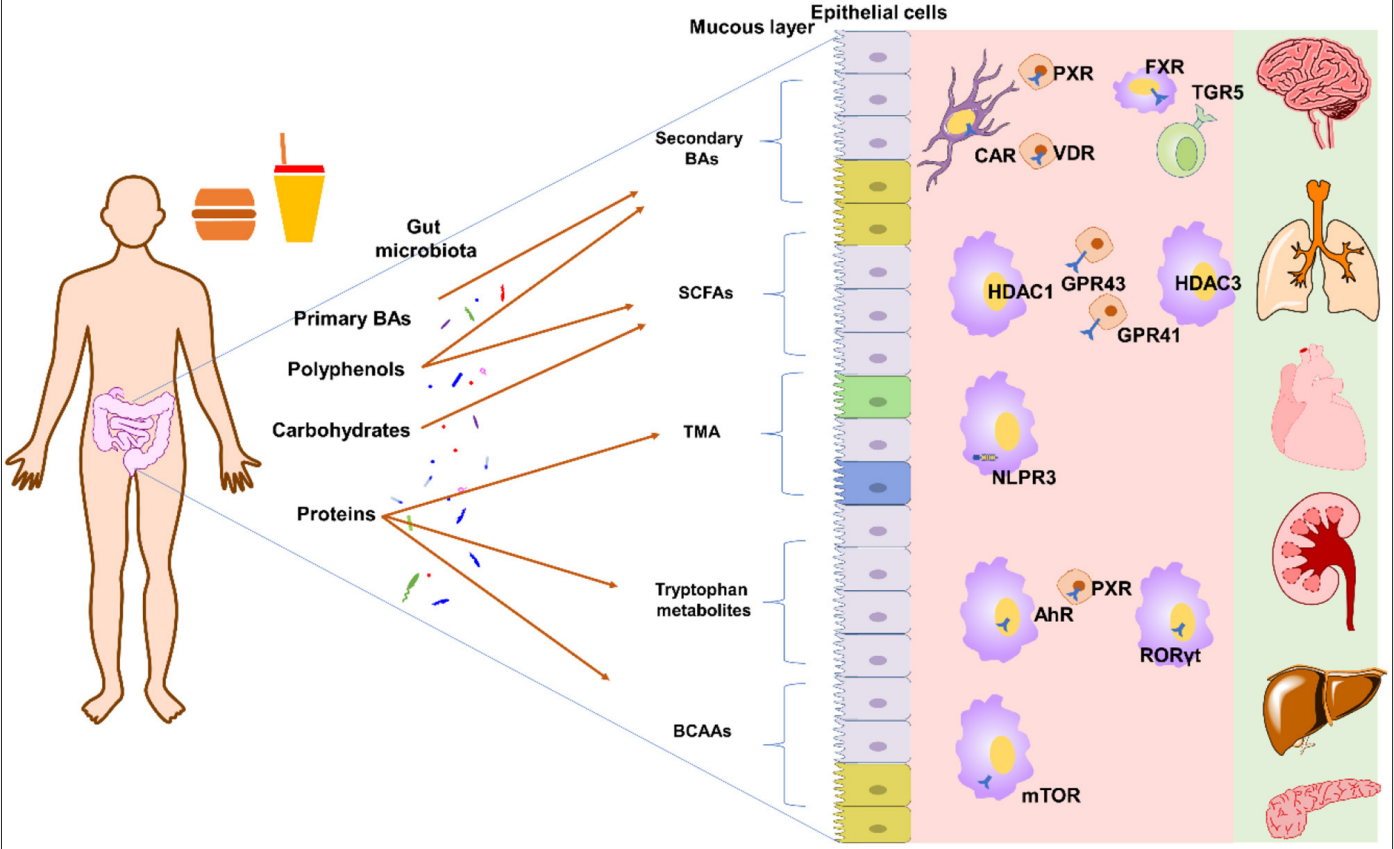
Typical gut microbiota derived metabolites and their targets. Primary BAs can by transformed into secondary BAs by bile salt hydrolase and 7α-dehydroxylase[[Inline Image]]. Polyphenols can modulate the composition of gut microbiota to affect SCFAs production or be directed degraded into SCFAs. Polyphenols can also affect 7α-dehydroxylase activity to influence BA pool. Carbohydrates can be directly fermented into SCFAs. Proteins can be degraded into peptides and amino acids, which can further be transformed into TMA, tryptophan metabolites, and others. Please refer to main text for detailed metabolites and targets. AhR, aryl hydrocarbon receptor; BAs, bile acids; BCAAs, branched-chain amino acids; CAR, constitutive androstane receptor; FXR, farnesoid X receptor; GPR41, G protein-coupled receptor 41; GPR43, G protein-coupled receptor 43; HDAC1, histone deacetylase 1; HDAC3, histone deacetylase 3; NLPR3, inflammasome NOD-like receptor protein 3; PXR, pregnane X receptor; RORγt, retinoid-related orphan receptor gamma-t; SCFAs, short-chain fatty acids; TMA, trimethylamine; TGR5, Takeda G-protein receptor 5; VDR, vitamin D3 receptor.

In general, dietary compounds in modulation of gut microbiota metabolites can be achieved by the following ways: (1) dietary compounds directly be metabolized into gut microbiota metabolites by gut microbiota; (2) dietary compounds modulate the gut microbiota composition and thus influence the metabolites-producing activity; (3) dietary compounds directly inhibiting enzymes responsible for production of gut microbiota metabolites; (4) regulating the expression or activity of hepatic enzymes responsible for host metabolism of gut microbiota metabolites; (5) a combination of these effects. The typical metabolites modulated by dietary compounds are SCFAs, bile acids, trimethylamine, branched-chain amino acids, tryptophan and indole metabolites (Figure 1).

### SCFAs

SCFAs are a group of small organic monocarboxylic acids with one to six carbon atoms (55), and they are the most widely studied gut microbiota metabolites in recent years. SCFAs are comprised mostly of acetate (C2), propionate (C3), and butyrate (C4) with a molar rate of 60:20:20 in gastrointestinal tract (95%) (56, 57). After the production, SCFAs can be readily absorbed by colonocytes *via* hydrogen-coupled monocarboxylate transporters and sodium-coupled monocarboxylate transporters, with only about 5–10 % of the SCFAs can be detected in feces (58, 59). The concentration gradient of SCFAs falls from the gut lumen to the periphery tissues and organs because of the consumption of butyrate at the epithelium, propionate at the liver and acetate in the periphery (60). After absorption, part of SCFAs will be directly utilized as energy resource for gluconeogenesis and lipid synthesis, while the remaining SCFAs play important roles in regulating various host biological responses such as inflammation and oxidative stress. SCFAs can activate several G protein-coupled receptors (GPCRs) directly, such as GPR43 (free fatty acid receptor 2, FFAR2) and GPR41 (FFAR3), GPR109a/HCAR2 (hydrocarboxylic acid receptor) and GPR164, and thus regulate energy metabolism, inflammation, oxidative stress, and other reactions (16, 61). SCFAs can also inhibit nuclear class I histone deacetylases (HDACs) including HDAC1 and HDAC3, and thus end up with regulation of inflammatory signaling pathways (16, 62). Under physiological condition, SCFAs can regulate gut microbiota composition, gut barrier, gut hormone, appetite, energy homeostasis, and immune function, and circadian clocks (18). On the contrary, disturbance of SCFAs is associated with a group of diseases such as diabetes, obesity, non-alcoholic fatty liver disease, hypertension, ulcerative colitis, Parkinson's disease, and colorectal cancer (18, 63).

Dietary compounds can directly modulate the production of SCFAs by serving as the resource for SCFAs synthesis. Non-digestible dietary carbohydrates such as fibers that escape the digestion and absorption of small intestine can be partially or completely fermented by gut microbiota and produce SCFAs. During fermentation, acetate can be produced by acetyl-CoA pathway or *via* the Wood-Ljungdahl pathway under the action of bacteria such as *Bifidobacterium* spp., *Lactobacillus* spp., and *Akkermansia muciniphila* (64). Propionate can be produced by succinate pathway, propanediol pathway, or acrylate pathway under the action of bacteria such as *Megasphaera elsdenii, Veillonella* spp. and *Roseburia inulinivorans* (65). Butyrate can be produced by butyryl-CoA:acetate CoA-transferase routes or phosphotransbutyrylase/butyrate kinase routes under the action of bacteria such as *Eubacterium rectale*, and *Faecalibacterium prausnitzii* (64, 65). In addition to carbohydrates, proteins or peptides that contain branched-chain amino acids and escape the digestion and absorption of small intestine can be fermented to SCFAs as well. However, the SCFAs produced from proteins and peptides are typically branched-chain fatty acids, such as 2-methylbutyrate, isobutyrate, and iso-valerate (66). Polyphenols can affect the SCFAs production as well. For example, the sugar moiety of quercetin-3-glucoside can be fermented to formate and acetate by *Enterococcus casseliflavus*, and the resulting quercetin can be further degraded into 3,4-dihydroxyphenylacetic acid, phloroglucinol, butyrate and acetate by *Eubacterium ramulus* (67).

The type of food and the intake amount can affect the types and amounts of SCFAs significantly. Dietary fiber, according to the definition by CODEX Alimentarius Commission, are carbohydrate polymers with ten or more monomeric units that are not hydrolyzed by the endogenous enzymes in the small intestine of humans (68). In animal models, the addition of fiber can lead to increase of cecal SCFAs in comparison with control diets. However, no significant linear correlation between fiber intake amount and SCFA concentrations can be observed (69). The study of SCFA concentrations in human is mainly carried by measuring the fecal SCFAs because of the convenience of the sampling method. However, concentrations of fecal SCFAs reflect little information on actual intestinal SCFA concentrations since SCFAs are readily absorbed by host after production. Therefore, *in vitro* fermentation is widely used to study the relationship between diet and SCFAs, and it was found out that different fiber showed significant difference in production of SCFAs (56). Specifically, the physical form and chemical properties of fibers including monosaccharide compositions, linkage of monosaccharide, molecular size and arrangements of the sugars are important factors influencing the production of SCFAs (70). For example, *in vitro* fermentation of polysaccharide from the seeds of *Plantago asiatica* L. showed that acetic and butyric acids are produced mainly due to the fermentation of aldehydes and xylose, whereas propionic acid is mainly generated by fermentation of arabinose and xylose (71). *Flammulina velutipes* polysaccharide with the monosaccharide composition at the ratio of glucose: galactose: mannose = 60.66:19.96:19.38 can increase the levels of isobutyric and butyric acid (72). On the contrary, the *Flammulina velutipes* polysaccharide with the monosaccharide composition at the ratio of mannose: glucose: xylose: arabinose: fucose = 6.6:27.8:18:1.5:5.2 increased the levels of acetic, propanoic and butyric acids (73). Because SCFAs play important roles in health and the type of food can affect the production of SCFAs, modulation of SCFAs by diet has been suggested to be a good approach to maintain health and treat diseases. In Table 2, we have listed the common dietary polysaccharides on SCFAs.

**Table 2:** Effects of dietary polysaccharides on SCFAs.

**Source**	**Models**	**Effects on gut microbiota**	**Effects on specific SCFAs**	**References**
Fucosylated chondroitin sulfate from sea cucumber *Stichopus chloronotus*	*In vitro* fermentation with human feces	Increased *Megamonas, Bacteroides, Parabacteroides, Prevotella, Fusobacterium*, and *Faecalibacterium*; decreased *Clostridium_XlVa, Bifidobacterium, Dialister, etc*.	Increased acetic acid, isobutyric acid, and isovaleric acid	(74)
Polysaccharides from *Gracilaria rubra*	*In vitro* fermentation with human feces	Increased *Bacteroidetes*; decreased *Firmicutes*	Increased acetic acid, propionic acid, and isobutyric acid	(75)
Polysaccharides from *Polygonatum kingianum*	High fat diet-induced obese rats	Increased *Bacteroides, Bifidobacterium*, and *Streptococcu*; inhibited *Lactobacillus* and *Psychrobacter*	Increased acetic acid, and propionic acid	(76)
Water-soluble polysaccharide from wild morels	Normal and cyclophosphamide (CP)-treated mice	Increased *Lachnospiraceae* in normal mice; increased *Ruminococcaceae* in CP -treated mice	Increased acetate, propionate, butyrate, and valerate in CP-treat mice	(77)
Polysaccharides from purple sweet potato	Normal and cyclophosphamide (CTX) treated mice	Increased *Bacteroidetes, Lachnospiraceae* and *Oscillospira*, and decreased *Firmicutes, Alcaligenaceae* and *Sutterella* in normal mice; increased *Bacteroidetes*, etc., decreased *Alcaligenaceae*, etc., in CTX-treated mice	Increased acetic acid, propionic acid and butyric acid in normal mice	(78)
Insoluble polysaccharide from the sclerotium of *Poria cocos*	*ob/ob* mice	Increased *Bacteroides, Lachnospiracea, Alloprevotella, Parabacteroides, Clostridum IV*, and *Ruminococcus*; decreased *Megamonas* and *Proteus*	Increased butyrate	(79)
Polysaccharides from blackberry	*In vitro* fermentation with human feces	Increased *Bacteroides, Parabacteroides, Prevotella*; decreased *Dorea, Blautia, Enterobacteriaceae, Coprococcus* and *Sutterella*	Increased acetic acid, propionic acid, butyric acid, isovaleric acid, valeric acid	(80)
Polysaccharides from *Tuber indicum*	Exhaustive swimming-induced fatigue mice	Increased *Porphyromonadaceae, Bacteroidetes*, etc.; decreased *Firmicutes, Proteobacteria, Ruminococcaceae, Helicobacteraceae*, etc.	Increased butyric acid.	(81)
Pumpkin polysaccharide	High-fat diet-induced T2DM rats	Increased *Alcaligenaceae, Prevotella, Sutterella, Burkholderiales, Deltaproteobacteria, Betaproteobacteria* and *Bilophila*.	Increased butyric acid and isovaleric acid	(82)
Polysaccharides from green tea	High-fat diet and streptozotocin-induced T2DM rats and normal rats	Enriched *Lachnospira, Victivallis, Roseburia*, and *Fluviicola* in T2DM rats	Increased acetic acid, propionic acid, n-butyric acid, i-butyric acid, and n-valeric acid in normal rats	(83)
Mulberry leaf polysaccharide	Cyclophosphamide-treated mice	Increased *Bacteroidetes*; decreased *Firmicutes, Butyricimonas* and *Eubacterium*.	Increased acetic acid, propionic acid, and n-butyric acid	(84)
Flaxseed polysaccharide	High-fat-diet-induced metabolic syndrome	Increased *Akkermansia* and *Bifidobacterium*; decreased *Oscillospira* and *Odoribacteraceae*.	Increased propionic acid and butyric acid	(85)
Tamarind seed polysaccharide	*In vitro* fermentation with human feces	Increased *Parabacteroides, Lactobacillus, Prevotella* and *Faecalibacterium*; decreased *Escherichia-Shigell* and *Dorea*	Increased propionic acid and butyric acid	(86)
Polysaccharide from *Artocarpus heterophyllus* Lam. pulp	Normal mice	Increased *Firmicutes, Proteobacteria, Cyanobacteria*; decreased *Bacteroidetes*	Increased acetic acid, propionic acid, n-butyric acid	(87)
*Spirulina platensis* crude polysaccharides	High-sucrose and high-fat diet-fed rats	Increased *Bacteroides, Corynebacterium, Alloprevotella, Paraprevotella, Flavonifractor*, etc.; decreased *Phascolarctobacterium, ifidobacterium, Lactobacillus* and *Romboutsia*	Increased butyric acid	(88)
Polysaccharide from *Schisandra chinensis*	Dextran sodium sulfate-induced ulcerative colitis mice	Increased *Alloprevotella, Saccharibacteria, Bacteroidetes*, etc.; decreased *Anaerotruncus*	Increased propionic acid, butyric acid and valeric acid	(89)
Polysaccharide from *Caulerpa lentillifera*	Cytoxan-induced immunosuppressed mice	Increased *Ruminococcaceae, Lactobacillus, Coriobacteriaceae, Helicobacter*, and *Clostridium_XVIII*; decreased *Bacteroides, Barnesiella* and *Lachnospiraceae*	Increased acetate	(90)
Polysaccharides from Rapeseed	High-fat diet-induced obesity	Increased *Blautia, Dorea, Akkermansia*; inhibited *Ruminococcaceae*	Decreased propionate and butyrate	(91)
Polysaccharides from *Solanum nigrum* L. (S1 and S2)	*In vitro* fermentation with human feces	Both polysaccharides increased *Faecalibacterium, Roseburia, Anaerostipes* spp.	S1 and S2 increased propionic acid, butyric acid, isobutyric acid, valeric acid and isovaleric acid; S2 also increased acetic acid and succinic acid	(92)
*Ficus carica* polysaccharide	Dextran sodium sulfate-induced colitis	Increased S24-7, *Bacteroides*, and *Coprococus*; decreased *Escherichia* and *Clostridium*	Increased acetate and butyrate	(93)
Depolymerized RG-I-enriched pectin from citrus segment membranes	Normal mice	DWRP: increased *Bifidobacterium* spp., *Lactobacillus* spp., *Faecalibaculum* spp. etc. WRP: increased *Bacteroides* spp*., Ruminococcus* spp, *Butyricicoccus* spp.etc.	Increased total SCFAs	(94)
Polysaccharides from Fuzhuan brick tea	*In vitro* fermentation with human feces	Increased *Prevotella* and *Bacteroides*; decreased *Firmicutes*	Increased lactic, acetic, propionic acids	(95)
Polysaccharides from bee collected pollen of Chinese wolfberry	*In vitro* fermentation with human feces	Increased *Prevotella, Dialister, Faecalibacterium Megamonas, Alloprevotella*; decreased *Bacteroides, Phascolarctobacterium, Clostridium XlVa, Parabacteroides*, etc.	Increased acetic and propionic acids	(96)
Apple polysaccharide	High-fat diet-fed rats	Increased *Bacteroidetes* and *Lactobacillus*; decreased *Firmicutes, Fusbacterium*.	Increased acetic acid and isobutyric acid	(97)
Polysaccharides from *Laminaria japonica*	High-fat diet-fed mice	Increased *Bacteroides* spp. and *Ruminococcaceae* spp.; decreased *Pseudomonas* spp., *Lachnoclostridium* spp.	Increased fecal butyric acid	(98)
*Oudemansiella radicata* polysaccharides	*In vitro* fermentation with human feces	Increased *Bacteroidales* and *Parabacteroides*	Increased acetic acid, propionic acid	(99)
Polysaccharides from *Flammulina velutipes*	*In vitro* fermentation with human feces	Increased *Bifidobacteriaceae* and *Bacteroidaceae*; decreased *Lachnospiraceae* and *Enterococcaceae*.	Increased acetate, propionate, butyrate, isobutyrate, valerate, isovalerate	(100)
Polysaccharide from oyster	*In vitro* fermentation with human feces	Increased *Bacteroides, Prevotella, Faecalibacterium, Parabacteroides, Blautia, Agathobacter*, etc., decreased *Escherichia-Shigella, Lactobacillus*, and *Bifidobacterium*	Increased acetic acid, propionic acid, and n-butyric acid	(101)
Polysaccharide from *Craterellus cornucopioides*	*In vitro* fermentation with human feces	Increased *Bacteroides, Parabacteroides, Citrobacter, Phascolarctobacterium*; decreased *Dorea, Gemmiger, Bifidobacterium*	Increased acetic, propionic and n-butyric acids	(102)
Jinxiang garlic polysaccharides	Dextran sulfate sodium-induced colitis mice	Increased *Muribaculacea*; decreased *Mucispirillum, Helicobacter, Bacteroides*.	Increased acetic acid and propionic acid	(103)
Polysaccharide from Mung bean skin	Normal mice	Increased *Firmicutes, Bacteroidetes, Clostridium* and decreased *TM7*	Increased acetic acid, propionic acid and butyric acid	(104)

In recent years, a large number of animal and clinical studies have carried to investigate the supplement of dietary compounds on diseases, especially fiber. In mice, supplement of dietary fermentable fiber changed the composition of gut and lung microbiota, especially the ratio of Firmicutes to Bacteroidetes. In addition, mice received a high-fiber diet showed increased circulating SCFAs and were protected from allergic lung inflammation (105). Compared with the animal studies, the clinical studies on supplement of dietary compounds have received more attentions. In a randomized clinical study, supplement of specifically designed isoenergetic diets showed that a high-fiber diet can change the composition of the gut microbiota and improve glucose homeostasis in patients with type 2 diabetes mellitus (T2DM). In addition, the high-fiber diet can selectively elevate bacteria producing SCFAs and the levels of bacteria producing SCFAs correlated positively with hemoglobin A1c levels in participants with T2DM (106). The results suggested that targeted promotion of bacteria producing SCFAs can be effective to treat T2DM in clinic. Considering that carbohydrates such as fibers can be fermented and transformed into SCFAs, it is no wonder that dietary supplement of these compounds can increase circulating SCFAs and can be conducive to amelioration of diseases. In fact, in addition to fermentable carbohydrates, many other dietary compounds especially polyphenol can increase SCFAs as well by changing the levels of bacteria producing SCFAs (107). For example, a major bioactive compound from green tea epigallocatechin-3-gallate protected the mice from colitis by increasing abundance of SCFAs-producing bacteria such as *Akkermansia* and levels of SCFAs (108). The mounting evidence suggested that manipulating gut bacterial SCFAs by dietary compounds can be useful to treat diseases.

### Bile acids

Bile acids (BAs) are a group of acidic, amphipathic, and water-soluble metabolites with a steroid structure that are originally produced from cholesterol in the hepatocytes. The primary BAs are synthesized mainly by classic pathway, in which 7α-hydroxylase acts as the rate-limiting enzyme, and to a lesser extent by alternative pathway (109). The main BAs synthesized in human are chenodeoxycholic acid and cholic acid whereas rodents can also produce muricholic acids, and thus BAs show evident species-specific differences (109). After the production, BAs can conjugate with taurine or glycine to form bile salts in the liver (110). When triggered by food intake, BAs are released into duodenum to exert their detergent-like activity to facilitate absorption of intestinal lipids. Meanwhile, most of the BAs can be reabsorbed and delivered back to liver, a process that is also known as enterohepatic circulation, to maintain the BA pool (111). For the unreabsorbed BAs, they can be deconjugated by gut microbiota under the catalysis of bile salt hydrolase, and further transformed into secondary BAs by 7α-dehydroxylase (20). A part of secondary BAs can also be absorbed from gut and get enriched through enterohepatic circulation, and thus may acting on hosts as important molecules to regulate the health and disease (20). The BA receptors include Takeda G-protein receptor 5 (TGR5, also known as G-protein-coupled BA receptor, GPBAR1), farnesoid X receptor (FXR), vitamin D3 receptor, pregnane X receptor/steroid and xenobiotic-sensing receptor, constitutive androstane receptor and others (19). By acting on these targets, BAs play important roles in regulation of lipids, amino acids, glucose metabolism and are implicated in a number of diseases such as obesity, insulin resistance, liver cirrhosis, non-alcoholic steatohepatitis and colitis (19). Worth notice is that BA receptors exhibit different affinity for different BAs. For example, TGR5 can be activated by BAs with the order of potency lithocholic acid > deoxycholic acid > chenodeoxycholic acid > cholic acid (including in both conjugated and unconjugated states) (112). This phenomenon indicates that variations of BA pool can result in perturbation of BA signaling in host, and this is confirmed by the fact that BA metabolism is disturbed in a number of diseases (113).

Given that dietary compounds can affect the composition of gut microbiota, dietary supplement may alter the gut microbial BA metabolism and thus further bring beneficial effects on hosts. In fact, a group of recent studies have continuously supported this idea. Curcumin is a compound that is extracted from turmeric, and is widely used as a dietary agent and traditional medicine for many years (114). In high-fat diet-induced obese wild type mice, curcumin treatment ameliorated obesity by reconstruction of gut microbiota composition and increasing of microbial derived secondary BAs including deoxycholic acid and lithocholic acid, two potent ligands for TGR5 (115). Moreover, the enhanced effects of curcumin on thermogenesis were eliminated inTGR5 knockout mice, which further confirmed that the effects of curcumin on obesity were achieved through modulation of microbial BA metabolism. Capsaicin, a naturally occurring alkaloid that derived from chillies, are widely used as food additives due to its hot pungent taste. In type 2 diabetic db/db mice, capsaicin remarkably improved glucose tolerance and insulin sensitivity, and this process was associated with decreasing the abundance of *Lactobacillus* and microbial bile salt hydrolase activity (116). The modulation of microbial abundance and enzyme activity further leaded to accumulation of tauro-β-muricholic acid, an antagonist of the FXR, and thus improved glucose metabolism and insulin sensitivity in diabetic mice (116). In another study by same group, capsaicin ameliorated high fat diet-induced obesity and adipose tissue accumulation, and this process was associated with the increase of *Bacteroides* abundance and 7α-dehydroxylase activity and the decrease of bile salt hydrolase activity. The modulation of microbial abundance and enzyme activity further leaded to the increase of lithocholic acid, an agonist of TGR5, and thus improved glucose metabolism and insulin sensitivity (117). These two studies showed similar pharmacological effects of capsaicin, yet the mechanisms were different. The reason may be that the animal models are different as one was leptin receptor-deficient diabetic mice whereas the other was high-fat diet-induced obese mice. Resveratrol, a natural polyphenol that is present largely in grapes and berries, improved glucose homeostasis by modulation of microbial BA metabolism and TGR5 in db/db mice (118). These studies suggested that the healthy effects of dietary compounds can be partly attribute to the modulation of gut microbial BA metabolism.

### Trimethylamine and trimethylamine N-oxide

Under the action of gut microbial trimethylamine (TMA) lyases, dietary quaternary amines that are not absorbed such as choline, betaine, phosphatidylcholine, and L-carnitine can be transformed into TMA (119). After absorption, TMA can be oxidized to trimethylamine N-oxide TMAO under the action of hepatic flavin-containing monooxygenases (FMOs) such as FMO1 and FMO3 (120). Increased TMAO then promotes oxidative stress and inflammation of endothelial cells, activates the inflammasome NOD-like receptor protein 3 and nuclear factor kappa B signaling in vascular smooth muscle cells, promotes the transformation of macrophages into foam cells, promotes platelet hyperreactivity, and alters cholesterol transport and bile acid synthesis (121). Correspondingly, high level of blood TMAO might contribute to heart disease, diabetes, atherosclerosis, and even cancer (122, 123).

Worth notice is that while a large number of epidemiologic research have showed strong association between the increased plasma TMAO concentrations and the risk of cardiovascular diseases, the roles of dietary compounds on final concentrations of TMAO in blood and the risk of diseases are still worth investigating as controversy remains over this topic. For example, a study on a longitudinal cohort of US men showed that intake of higher levels of red meat and choline was significantly associated with higher TMAO levels in participants with rich TMAO-producing bacteria especially *Alistipes shahii* but not in other participants (124). On the contrary, a study comparing the TMAO levels in patients with carotid artery atherosclerosis and healthy controls showed that there was no association between the level of TMAO and carotid atherosclerosis or cardiovascular death (125). In addition, fish-rich diets can lead to obvious increase of plasma TMAO; however, meta-analysis, cross-sectional and prospective studies showed that fish consumption is associated with lower risk of cardiovascular diseases (126). Furthermore, some studies even reported the beneficial effects of TMAO or TMA in cardiovascular diseases (119).

Nevertheless, in addition to the compounds such as choline and L-carnitine that can be metabolized to TMA directly, modulation of TMA and TMAO metabolism is still regarded as one important mechanism of other types of dietary compounds in regulation of cardiovascular diseases. For example, administration of *Lonicera caerulea* berry extract, which is rich in polyphenols, to high cholesterol-induced hypercholesterolemic male SD rats for 12 weeks attenuated serum dyslipidemia (127), and this process was associated with decreasing of serum TMAO levels. In mice supplemented with 1.3% carnitine, flavonoids from oolong tea and citrus peels remarkedly reduced plasma TMAO and aortic inflammation, and this process was associated with modulation of *Lactobacillus* and *Akkermansia* (128). In addition to crude extracts, pure compounds in diet can also modulate TMA and TMAO to modulate cardiovascular diseases. For example, oral resveratrol can attenuate TMAO-induced atherosclerosis in ApoE^−/−^ mice, and this process was associated with inhibiting of gut microbial TMA production (129). Besides, treatment with antibiotics abolished the decreasing effects of resveratrol on TMAO levels and the inhibiting effects on atherosclerosis.

### Branched-chain amino acids

Branched-chain amino acids (BCAAs), including leucine, isoleucine, and valine, are essential amino acids with branched aliphatic side chains. BCAAs play critical roles in nutrients metabolism such as muscle protein synthesis, glucose and lipid metabolism *via* phosphoinositide 3-kinase/protein kinase B/mammalian target of rapamycin signaling pathway (130). In contrast to the beneficial effects, evidence has indicated that BCAAs can contribute to a number of diseases such as insulin resistance, type 2 diabetes mellitus, cancer, and cardiovascular diseases (26, 130). Therefore, BCAAs has been suggested to be developed as biomarkers to predict the outcomes of diseases such as obesity, insulin resistance, and cardiovascular diseases (131). Although mammalians lack the enzymes needed for the *de novo* synthesis of BCAAs, gut microbiota contain rich enzymes responsible for synthesis of BCAAs (132). Many gut bacteria are capable of *de novo* synthesis of BCAAs, such as *Prevotella copri, Bacteroides vulgatus*, and *Clostridium clusters* (133). Since BCAAs can be degraded from food and synthesized by gut microbiota, both the daily supplementation of BCAAs-containing food and modulating the composition of gut microbiota can affect the circulating BCAAs and further affect the health and disease associated with BCAAs. For example, diet with specifically reduced BCAAs can reverse the diet-induced obesity and restore the glucose tolerance and insulin sensitivity (134).

In addition to the dietary proteins, peptides, and amino acids that can be directly metabolized to BCAAs by gut microbiota, other dietary compounds can also influence the levels of circulating BCAAs and thus attenuate diseases. For example, virgin olive oil and high-oleic acid peanut oil are dietary vegetable oil with a high monounsaturated fatty acid and can attenuate metabolic syndrome (135). When the two types of oils were fed to rats with a high-fat diet, the β-diversity and abundance of *Bifidobacterium* were increased (oleic acid peanut oil also decreased *Lachnospiraceae* and *Blautia*) and the levels of BCAAs were revised, suggesting of modulation of gut microbial BCAA metabolism as the mechanism of the two oils in attenuation of metabolic syndrome (135). As a favorite vegetable, *Luffa cylindrica* (L.) Roem (*luffa*) is a very common in daily diet, and it is rich in polyphenols, saponin, triterpenoids, flavonoids, and oleanolic acid (136). Dietary oral administration of *luffa* to diet-induced obese mice can reduce circulating BCAA levels and selectively decrease the relative abundances of bacteria such as *Enterortabdus* and *Butyricicoccus* positively correlated with BCAA levels (136). In addition, this effect on BCAA catabolism was not observed in antibiotic-treated obese mice. The results suggested that *luffa* ameliorated obesity by modulation of gut microbial BCAA metabolism. In another study, citrus polymethoxyflavones ameliorated high-fat diet-induced metabolic syndrome *via* regulation of gut microbial BCAA metabolism (137). Antibiotic treatment, fecal microbiome transplantation, and single bacterium gavage showed that *Bacteroides ovatus* was responsible for the reduction of BCAA levels and alleviation of metabolic syndrome (137).

### Tryptophan, indole, and indole derivatives

Tryptophan, an essential amino acid for human body, is an important metabolite that can significantly influence both the physiology and pathology of mammalians. In addition to the main role in protein synthesis, tryptophan in host cells can act as an important precursor for production of crucial metabolites following the kynurenine and serotonin pathways (28). In contrast, unabsorbed tryptophan in the colon can interact with gut microbiota and can be metabolized into indole and indole derivatives such as skatole and indole acrylic acid under the action of the tryptophanases, which are expressed in many Gram-negative and Gram-positive gut bacteria such as *Clostridium* spp., *Bacteroides* spp. and *Escherichia coli* (28). Many of the indole derivatives such as indole acrylic acid, indole-3-aldehyde, indole-3-propionic acid, indole-3-acetic acid, and indole-3-acetaldehyde can act as ligands for aryl hydrocarbon receptor (AhR), pregnane X receptor and retinoid-related orphan receptor gamma-t (29, 138). Among these targets, aryl hydrocarbon receptor plays important roles in intestinal homeostasis by acting on a group of cells, such as Th17 cells, macrophages, dendritic cells, neutrophils, and other cells (139). Correspondingly, microbial tryptophan metabolites can regulate the intestinal barrier integrity, epithelial proliferation, and intestinal resistance to pathogens (27, 28). Since intestinal barrier are directly associated with a number of diseases, tryptophan and tryptophan microbial metabolites have been demonstrated to be linked to a number of diseases such as ulcerative colitis, Crohn's disease, obesity, autism spectrum disorder, Alzheimer's disease, Parkinson's disease, and irritable bowel syndrome (28, 140, 141).

As microbial tryptophan metabolism is associated with host functions and diseases, it is no wonder that dietary tryptophan is associated with health. In the aging mice, a diet containing 0.4% tryptophan significantly attenuated the inflammation and oxidative stress *via* increasing the colon indoles and the relative abundance of *Akkermansia* (142). Similarly, in non-obese diabetic mice expressing DQ8, a tryptophan-rich diet decreased gluten immunopathology *via* modulation of gut microbiota composition and enhancing the production of AhR ligands such as indole-3-aldehyde and indole3-lactic acid (143). On the contrary, in an aged mouse model, tryptophan-deficient diets (0.1% tryptophan) showed an elevated levels of pro-inflammatory cytokines in comparison with those fed a normal (0.2% tryptophan) and a tryptophan-rich (1.25% tryptophan) diet (144). And the effect was associated with modulation of gut bacteria such as *Clostridium* spp., which is involved in microbial tryptophan metabolism (144).

In addition to the diet containing tryptophan, other types of dietary compounds can modulate the gut microbiota tryptophan metabolism as well. For example, turmeric polysaccharides can improve the pathological phenotype of colitis mice *via* increasing the relative abundance of gut bacteria associated with tryptophan metabolism such as *Lactobacillus* and increasing the levels of cecal indole-3-acetic acid (145). Similarly, Fuzhuan brick tea polysaccharide ameliorated the ulcerative colitis of mice *via* promoting the proliferation of beneficial bacteria such as *Akkermansia* and increasing of fecal indole-3-acetaldehyde and indole-3-acetic acid (146). 1-Deoxynojirimycin, a major bioactive compound from functional food mulberry leaves, can ameliorate hyperlipidemia *via* increasing of *Akkermansia* and *Clostridium* and enhancing the microbial production of indole-3-propionic acid (147). Besides the animal-based studies, clinical studies have also suggested the beneficial effects of the other types of dietary compounds on microbial tryptophan metabolism. For instance, in a randomized, controlled, crossover trial, a polyphenol-rich diet significantly increased the microbial tryptophan metabolite indole 3-propionic acid in older people with normal renal function, and this effect was associated with shift of bacteria Bacteroidales and Clostridiales (148).

### Gases

Gut microbiota can produce gases (H_2_S, H_2_, CO_2_, CH_4_, NO) (6, 35). CH_4_ has significant effect on gastrointestinal tract as it is associated with constipation and can slow the colon motility (149). NO plays important roles in neuronal communication, modulation of blood vessels and immune response, and is possibly participated in diseases such as stroke and Parkinson's disease (150). While high concentration of H_2_S is generally believed to be toxic to hosts by inducing mucus disruption and inflammation, low levels H_2_S can be beneficial to hosts by stabilizing mucus layer, inhibiting adherence of bacteria to the epithelium, preventing the invasive bacteria, and reducing inflammation and tissue injury (151).

CO_2_ is mainly produced in the stomach whereas H_2_, CH_4_, CO_2_ and H_2_S are mainly generated in the small intestine and colon (31). The gases H_2_ and CO_2_ are produced by fermentation of undigested carbohydrates and, to a much lesser extent, by dietary or hosts-released proteins, whereas the gas CH_4_ is produced with the help of archaea in the colon by metabolism of CO_2_ and H_2_ (152). H_2_S is generated by fermentation of sulfur-containing proteins with the help of bacteria that are capable of reducing sulfates and sulfites (153). In mammalian cells, H_2_S can also be generated from L-cysteine, thio sulfate and homocysteine with the help of enzymes such as cystathionine β-and γ-lyase, aspartate aminotransferase and 3-mercaptopyruvate sulfurtransferase (154, 155). Since chemical constituents and fermentable substrates are affected by dietary intake, the diet can change the production of gases. For instance, cellulose and corn bran can improve the production of H_2_, whereas the production of CH_4_ was not affected (156, 157). In healthy volunteers, a diet with high levels of short-chain carbohydrates that are poorly absorbed and non-digestible in the small intestine leaded to an increase of H_2_ levels and a decrease of CH_4_ (158). In addition, consuming of two purified fibers xylans and pectin increased the levels of CH_4_, whereas lactulose did not show this effect (156, 159). Taken together, dietary compounds can be metabolized into gases and the types of diet can influence the gas production.

### Others

In Table 3, we have listed the non-polysaccharide dietary compounds and extracts on aforementioned gut microbiota-derived metabolites. In addition to these metabolites, gut microbiota can also produce other metabolites such as lipids (lipopolysaccharides, conjugated fatty acids, etc.), vitamins, ethanol, triphosadenine, organic acids (such as benzoate and hippurate), polysaccharide A, imidazole propionate, dipeptide aldehydes (35, 176–179). Among these metabolites, of special note are the metabolites produced from amino acids. In addition to tryptophan, other amino acids can also interact with gut microbiota and be transformed. Dietary tyrosine can be transformed into phenol, a molecule with possible roles in large bowel cancer and leukemia, under the action of gut bacterial-specific tyrosine phenol-lyase (180). Another study reported that tyrosine can be transformed into phenol and *p*-cresol under the action of tyrosine phenol-lyase and (or) hydroxyarylic acid decarboxylase, and *p*-hydroxyphenylacetate decarboxylase and (or) tyrosine lyase, respectively (181). *p*-cresol exhibits deleterious metabolic and genotoxic effects on colonic epithelial cells, and its level is associated with chronic kidney disease, cardiovascular disease, and autistic-like behaviors (182, 183). Phenylalanine can be transformed into phenylacetate under a group of enzymes such as phenylacetate-CoA ligase in bacteria such as *Escherichia coli* and *Pseudomonas putida* (184). Methionine can be metabolized into methanethiol, NH_3_, and 2-oxobutanoate under the action of methionine γ-lyase, an enzyme that exists in *Pseudomonas putida* and other bacteria (185). Gut microbiota can transform amino acid into polyamines such as agmatine, putrescine, spermidine, cadaverine. For example, lysine can be transformed into cadaverine through diaminopimelic acid route and the α-aminoadipic acid pathway (186). High concentration of cadaverine can be toxic and potentiate histamine toxicity and is associated with ulcerative colitis (179). Gut microbiota can also produce neuroactive compounds and neurotransmitters, such as γ-aminobutyrate (GABA), norepinephrine, dopamine, histamine, and serotonin, by catabolism of amino acids (187, 188). For example, glutamate can be transformed into γ-aminobutyrate *via* the enzyme glutamate decarboxylase and bacteria such as *Lactobacillus* spp. and *Bifidobacterium* spp. (189). These gut microbiota-derived neuroactive compounds and neurotransmitters play important roles in gut motility disorders, behavioral disorders, neurodegenerative diseases, cerebrovascular diseases, and neuroimmune-mediated disorders (190). Taken together, amino acids can be transformed into neuroactive compounds, sulfide-containing metabolites (H_2_S, methanethiol), aromatic compounds (phenol, *p*-cresol), and polyamines, under the action of gut microbiota.

**Table 3:** Non-polysaccharide dietary compounds and extracts in modulation of typical gut microbiota-derived metabolites.

**Metabolites modulated**	**Typical dietary compounds**	**Models**	**Gut microbiota involved**	**Effects on gut microbiota-associated metabolites**	**References**
SCFAs	Epigallocatechin-3-gallate	Bisphenol A-induced insulin resistance and microbial disturbance mice	Increased *Lachnospiraceae* and *Ruminococcaceae;* reduced *Firmicutes* and *Clostridia*	Increased acetic acid, butyric acid, and valeric acid	(160)
	Ginsenoside Rk3	Antibiotic-induced gut microbiota disturbance and low-grade inflammation mice	Enriched genera *Bacteroides, Alloprevotella* and *Blautia*; decreased *Firmicutes*/*Bacteroidetes* ratio	Increased acetic acid, propionic acid, butyric acid, isobutyric acid, valeric acid, and isovaleric acid	(161)
	Hydroxysafflor yellow A	High-fat diet-induced obese mice	Increased *Akkermansia, Romboutsia, Butyricimonas* and *Alloprevotella*; decreased *Firmicutes*/*Bacteroidetes* ratio	Increased acetic acid, propionic acid, and butyric acid	(54)
	Gallic acid	Dextran sulfate sodium salt-induced colitis rats	Increased *Bacteroides* and *rc4_4*; decreased *Enterobacteriaceae, Peptococcaceae, Turicibacteraceae*	Decreased acetate, propionate, iso-butyrate, and butyrate	(162)
	Chlorogenic acid	High-fat diet-induced obese rat	Decreased *Blautia, Sutterella*, and *Akkermansia*	Increased butyric acid	(163)
	Quercetin-3-glucoside	*In vitro* fermentation with human fecal suspensions and single bacteria	*Enterococcus casseliflavus* and *Eubacterium ramulus*	Butyrate, acetate, and formate were detected	(67)
	Green tea polyphenols	Antibiotic-induced gut microbiota disorder mice	Increased *Lactobacillus, Akkermansia, Blautia, Roseburia*, and *Eubacterium*, etc	Increased acetic acid and butyric acid	(164)
	Anthocyanins from fruits of *Lycium ruthenicum* Murray	Dextran sodium sulfate-induced colitis mice	Increased *Porphyromonadaceae, Rikenellaceae* and *Prevotellaceae*	Increased acetic, propionic, i-butyric acids	(165)
	Blueberry anthocyanin-rich extract	In high-fat and high-sucrose diet-induced obese mice	Decreased *Lachnospiraceae bacterium Choco86, Blautia* sp. *N6H1-15*, and *Ruminococcus torques*	Decreased valeric acid, isobutyric acid, and isovaleric acid	(166)
	Lychee (*Litchi chinensis* Sonn.) pulp phenolics	Dextran sodium sulfate-induced colitis mice	Increased *Akkermansia, Lactobacillus, Coprococcus*, and *Bacteroides uniformis*; decreased *Enterococcus* and *Aggregatibacter*	Increased propionic, n-valeric, and iso-valeric acids	(167)
	Polyphenols from *Camellia japonica* bee pollen	Hyperuricemia mice induced by potassium oxonate	Increased *Bacteroidetes, Actinobacteria* and *Proteobacteria*; decreased *Firmicutes*	Increased acetic acid and butyric acid	(168)
BAs	Capsaicin	High-fat diet-induced obese mice	Increaseed *Bifidobacterium, Bacteroides, Akkermansia muciniphila*, etc	Increased lithocholic acid	(117)
	Theabrownin	High-fat diet-induced obese mice	Increased species richness, decreased *Firmicutes*/*Bacteroidetes* ratio	Decreased deoxycholic acid, 23-nordeoxycholic acid, taurodeoxycholic acid; increased lithocholic acid	(169)
	Dihydromyricetin	Dextran sulfate sodium-induced colitis mice	Increased *Lactobacillaceae*; decreased *Lachnospiraceae, Peptostreptococcaceae*, and *Streptococcaceae*	Increased α-muricholic acid and; decreased isolithocholic acid, and taurohyodeoxycholic acid	(170)
	Epigallocatechin-3-gallate	High-fat diet-induced dysbiosis	Increased *Adlercreutzia, Akkermansia, Allobaculum*; decreased *Desulfovibrionaceae*	Decreased deoxycholic acid	(171)
	Apple polyphenols	Dextran sulfate sodium-induced ulcerative colitis	Increased *Verrucomicrobia, Bacteroides* and *Akkermansia*; decreased *Bacterodetes*	Decreased hyodeoxycholic acid	(172)
TMA (TMAO)	Allicin	Healthy participants, *L*-carnitine-fed mice, ApoE^−/−^ mice	Increased *Akkermansia* and *Faecalibacterium prausnitzii*	Decreased TMA and TMAO	(173)
	Baicalin	Repeated cerebral ischemia-reperfusion injury mice	Increased *Citromicrobium_sp_WPS32* and *Eubacterium_sp_CAG_86*; decreased *Lactobacillus_plantarum*	Decreased TMA and TMAO	(174)
BCAAs	Citrus polymethoxyflavones	Metabolic syndrome induced by high-fat diet	Enriched the commensal bacterium *Bacteroides ovatus*	Reduced the levels of valine, leucine, isoleucine	(137)
Tryptophan and indole derivatives	Ginsenoside Rg1	Ulcerative colitis mice induced by dextran sulfate sodium	Increased *Lactobacillus, Allobaculum*, and *Akkermansia*; decreased *Odoribacter, Clostridia_UCG-014, Bacteroides*, and *Turicibacter*	Increased indole-3-formaldehyde, 3-indolepropionic acid and decreased tryptophan	(175)

## Perspectives

Because of the importance of gut microbiota metabolites in health and disease, regulation of gut microbiota metabolites by diets has received much attention in recent years. However, gut microbiota itself show great intra- and interindividual variations (1) and thus the extent of the response of gut microbiota metabolites in one person to a diet might be different from the response of another person. In this background, the concept precision nutrition or personalized nutrition has been raised in recent years following the initiative of precision medicine (191). Currently, targeted manipulating of gut microbiota has become an important approach to achieve precision medicine (43). Similar to precision medicine, precision nutrition aims to identify key characteristics of gut microbiota that can predict the response of an individual to specific dietary components, and then design of a diet that is conductive to health. In a proof-of-principle study, analysis of three cohorts of obese adults from Belgium, Finland, and Britain showed that Clostridial species were indicative of the response of gut microbiota to diet, which in turn predicted the hosts' cholesterol response (192). In another study, a machine-learning approach integrating gut microbiota, blood parameters, dietary habits, anthropometrics, and physical activity showed that this approach can accurately predicts individual postprandial glycemic response after real-life meals (193). However, these studies did not correlate the levels of gut microbiota metabolites with diets and clinical outcomes, further studies are needed to identify the metabolites that are capable of predicting clinical outcomes.

After intake of a specific diet, the change of gut bacteria and gut microbiota metabolites may not be limited to a specific bacterium or metabolites. For example, in a mice model of inflammatory bowel disease, ketogenic diet altered gut bacteria such as *Proteobacteria, Enterobacteriaceae, Escherichia-Shigella* and changed the gut microbiota metabolites such as stearic acid, arachidic acid, and erucic acid (194). In fact, many of the changed bacteria and metabolites only show associative relationship with the change of phenotypes, and the key bacteria and metabolites with causal relationship must be determined. This idea is supported by the recent idea of keystone taxa, which drive the community composition and function of microbiota irrespective of their abundance (195). Therefore, screening of the key bacteria and gut microbiota metabolites responsible for the phenotypes is necessary. To achieve this aim, the combination of *in vivo* and *in vitro* experiments, and a variety of advanced technologies can be used. In this case, animal such as germ-free animal, gnotobiotic animal, antibiotic-treated animal and fecal bacteria culturing can be adopted to screening key bacteria. Targeted and untargeted metabolomics combined with oral supplementation or intravenous injection can be adopted to identify the key metabolites.

## Conclusion

Gut microbiota-derived metabolites such as SCFAs and BAs play important roles in maintenance of host homeostasis and development of diseases. In the colon, the dietary compounds that pass small intestine can interact with gut microbiota and thus modulate the gut microbiota metabolites. In general, these metabolites can be grouped into 3 types: (I) metabolites that are produced directly from dietary compounds by gut microbiota; (II) metabolites that are originally synthesis by hosts and chemically transformed by gut microbiota; (III) metabolites that are produced by gut microbiota *de novo*. Depending on the types of dietary compounds, the gut microbiota metabolites affected include, but not limited to, SCFAs, BAs, TMA, BCAAs, and tryptophan metabolites. Because some dietary compounds can be metabolized to a specific group of metabolites under the action of particular bacteria, personalized diets can be applied for targeted manipulation of gut microbiota metabolites. In addition, a specific diet can influence multiple gut bacteria and metabolites, thus, identification of key bacteria and metabolites responsible to the healthy effects of diets are necessary.

## Author contributions

Conceptualization and funding acquisition: WF and CP. Writing—original draft preparation: WF. Writing—review and editing: JL, HC, DZ, and YT. Supervision and project administration: CP and YT. All authors have read and agreed to the published version of the manuscript.

## Funding

This work was supported by the National Natural Science Foundation of China (Nos. 82104409, 81891012, 81891010, and U19A2010), Science and Technology Ministry of China (2108ZX09721001–008), China Postdoctoral Science Foundation (No. 2021M690490, China), Sichuan Science and Technology Program (Nos. 2021YJ0466 and 2022C001, China), National Interdisciplinary Innovation Team of Traditional Chinese Medicine (ZYYCXTD-D-202209), and Xinglin Scholar Plan of Chengdu University of Traditional Chinese Medicine (BSH2020017).

## Conflict of interest

The authors declare that the research was conducted in the absence of any commercial or financial relationships that could be construed as a potential conflict of interest.

## Publisher's note

All claims expressed in this article are solely those of the authors and do not necessarily represent those of their affiliated organizations, or those of the publisher, the editors and the reviewers. Any product that may be evaluated in this article, or claim that may be made by its manufacturer, is not guaranteed or endorsed by the publisher.
